# Extraction Optimization and Characterization of Antioxidant Polysaccharides From *Suillus bovinus*

**DOI:** 10.1002/fsn3.70995

**Published:** 2025-09-19

**Authors:** Zhuang Li, Shuting Fan, Xueqi Wang, Li‐an Wang, Jianhua Lv, Jinxiu Zhang

**Affiliations:** ^1^ College of Life Sciences Hebei Normal University Shijiazhuang People's Republic of China; ^2^ Gaobeidian No. 1 Middle School Baoding People's Republic of China

**Keywords:** antioxidant activity, polysaccharide, structural analysis, *Suillus bovinus*

## Abstract

A three‐variable, three‐level Box–Behnken design combined with response surface methodology (BBD‐RSM), based on single‐factor experiments, was employed to optimize the extraction parameters of crude polysaccharides (SubPs) from *Suillus bovinus*. SubPs were purified using a DEAE‐52 cellulose column and a Sephadex G‐100 chromatography column, yielding four homogeneous fractions (S‐1, S‐2, Y‐1, Y‐2) with molecular weights from 47.34 to 97.07 kDa. Among these fractions, Y‐1 exhibited superior antioxidant activity, demonstrating a 34.73% ± 1.31% reduction in reactive oxygen species (ROS) levels at 50 μg/mL compared to the tert‐butyl hydroperoxide (*t*‐BHP)‐treated model group in HepG2 cells, along with enhanced activities of superoxide dismutase (SOD), catalase (CAT), and glutathione peroxidase (GSH‐Px), as well as reduced malondialdehyde (MDA) content. The chemical structure of Y‐1 was further characterized through monosaccharide composition analysis, methylation analysis, and nuclear magnetic resonance (NMR) spectroscopy. These findings provide structural insights into Y‐1 and highlight its potential as a natural antioxidant.

## Graphical abstract

BBD‐RSM optimized bioactive polysaccharide extraction from *Suillus bovinus*. Y‐1 from S. bovinus reduced ROS, boosted SOD/CAT/GSH‐Px, and lowered MDA in HepG2 cells. Y‐1 has a unique heteropolysaccharide structure.

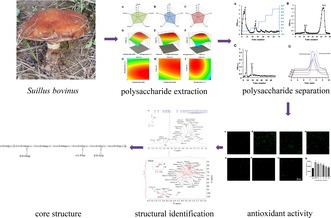

## 1 Introduction

Polysaccharides extracted from fungi are often identified as biological response modifiers due to their antioxidative activities. Various mushroom‐derived polysaccharides with antioxidant activity have been reported, with the most extensively studied being those from Ganodermataceae (Tian et al. 2012). Additionally, antioxidative polysaccharides have been characterized in *Inonotus obliquus* (Ma et al. 2013), *Paxillus involutus* (Liu et al. 2018), *Hericium erinaceus* (Zaitseva et al. 2024), *Pleurotus ferulae* (Muhaxi et al. 2023), *Agrocybe aegerita* (Du et al. 2022), and *Coprinus comatus* (Wang et al. 2021).

The genus *Suillus*, commonly known as slippery jacks, comprises the initial colonizers of pine seedlings (Sun et al. 2023). Its distribution aligns with the natural range of Pinaceae in the Northern Hemisphere (Ruiz‐Diez et al. 2006). *Suillus* is crucial in conifer establishment and includes medium to large terrestrial boletes. Most species within this genus feature a veil, and the cap is often viscid or slimy; some also possess glandular‐dotted stems (Heinke et al. 2014). *S. bovinus* produces edible fruit bodies with a high economic value (Feng et al. 2022) and is considered a delectable food source, rich in protein, vitamins, essential amino acids, and other nutrients. Additionally, they contain anti‐cancer compounds and exhibit anti‐tumor, antioxidant, and anti‐inflammatory properties, thereby possessing both medicinal and culinary significance (Juan 2007; Zhang et al. 2024). The antioxidant capacity of *S. bovinus* was evaluated using the trolox equivalent antioxidant capacity (TEAC) assay and ferric reducing antioxidant power (FRAP) assay, indicating its potential as an important dietary source of natural antioxidants due to its high total phenolic content (Guo et al. 2012).

At present, the majority of studies on *S. bovinus* focus on its interactions with the environment as an ectomycorrhizal fungus (Kothamasi et al. 2019; Shirakawa et al. 2019; Sousa et al. 2012). Polysaccharides play a critical role among biologically active components due to their significant medical and pharmaceutical properties (Sivanesan et al. 2022), including antioxidant, immunostimulatory, neuroprotective, and antitumor effects (Du et al. 2022; Ghosh et al. 2021; Jiang et al. 2022; Niu et al. 2021). The polysaccharides from *S. bovinus* have demonstrated antioxidant capacities, and further analysis is required to determine the monosaccharide composition of the active fraction (Guo et al. 2012; Zhao et al. 2024). Consistent with these findings, we aim to optimize the extraction conditions for crude polysaccharides and identify the specific components responsible for this antioxidant activity. This study seeks to establish a foundation for the development and utilization of polysaccharides from *S. bovinus*.

## 2 Materials and Methods

### 2.1 Materials and Reagents

*S. bovinus* was purchased from a local market in Shijiazhuang, China, and was identified using internal transcribed spacer (ITS) sequencing. DEAE‐52 cellulose and Sephadex G‐100 were obtained from Solarbio (Beijing, China). Dulbecco's modified eagle medium (DMEM) and fetal bovine serum (FBS) were purchased from Gibco (Shanghai, China). All other reagents used were analytical grade.

### 2.2 Single‐Factor Design for Crude Polysaccharide Extraction

The crude polysaccharides from the *S. bovinus* were extracted using distilled water at 80°C for 1 h with a liquid‐to‐solid ratio of 10 mL/g. After centrifugation and filtration, protein in the supernatant was removed using the Sevag method (chloroform/n‐butanol, 4:1, *v*/*v*) (Cao et al. 2024; Sevag and Maiweg 1934). The extraction solution was concentrated using a vacuum rotary evaporator and then precipitated by adding ethanol to achieve an 80% (*v*/*v*) final concentration, which was left overnight at 4°C. The precipitate was centrifuged and subsequently dissolved in distilled water, reprecipitated with 80% ethanol, and dried by vacuum freeze‐drying.

The extraction yield (%) was calculated as follows:

$$\begin{array}{cc} \text{Extraction yield (\%)}= & [\text{weight of dried crude polysaccharide (g)}/\text{weight of}\;\\ & S.\text{bovinus}\;\text{powder (g)}]\times \text{100\%} \end{array}$$

### 2.3 Experimental Design for Response Surface Methodology (RSM)

Three different extraction conditions were evaluated to assess their effects on polysaccharide yield: liquid‐to‐solid ratios ranging from 10 to 50 mL/g, extraction temperatures from 30°C to 70°C, and extraction times from 1 to 5 h.

Based on the results of the single‐factor tests, a three‐variable, three‐level, 17‐run Box–Behnken design (BBD) was employed to statistically optimize the extraction of SubPs (Tables S1 and S2). The experimental design was conducted using Design Expert software (Version 11). All experiments were performed in triplicate.

### 2.4 Purification of SubPs

The SubPs was extracted under optimal conditions and dried using vacuum freeze‐drying. One hundred milligrams of the dried crude polysaccharide was dissolved in deionized water and applied to a DEAE cellulose‐52 column (2.6 × 20 cm). The column was sequentially eluted with 0, 0.1, 0.3, 0.5, and 0.7 M NaCl at a flow rate of 2.0 mL/min. Eluted fractions (4 mL per tube) were collected, and the carbohydrate content of each fraction was determined using the phenol‐sulfuric acid method (DuBois et al. 1956; Wang et al. 2025). The main fractions eluted by 0 and 0.1 M NaCl were designated as Y and S, respectively. These fractions were further purified using a Sephadex G‐100 column (2.6 × 60 cm), eluting with 0.1 M NaCl at a flow rate of 0.2 mL/min to separate polysaccharide components based on their molecular weights.

### 2.5 Molecular Weight Measurement

Gel permeation chromatography (GPC) was performed to determine the molecular weight of the polysaccharides using Waters 1525 equipment. Specifically, a 30 mg sample was dissolved in 1 mL of KH_2_PO_4_ (0.02 M), filtered through a 0.22‐μm filter, and 10 μL of the filtrate was injected into a gel‐filtration chromatographic column (Aquagel‐OH MIXED‐H 7.8 × 300 mm, 8 μm). The mobile phase consisted of ultrapure water containing 0.2 M sodium nitrate and 0.01 M NaH_2_PO_4_. The column temperature was maintained at 30°C with a flow rate of 1 mL/min.

### 2.6 Cell Culture and Cell Viability Assay

HepG2 cells were cultured in DMEM supplemented with 10% fetal bovine serum at 37°C in a humidified atmosphere containing 5% CO_2_. Cell viability was assessed using the CCK‐8 assay as described previously (Zhang et al. 2020). Briefly, HepG2 cells were seeded at a density of 1 × 10^4^ cells/well in 96‐well plates and incubated for 24 h. Subsequently, the cells were treated with four polysaccharide components (0–50 μg/mL) for 6 h. Following this, the cells were exposed to 0.4 mM *t*‐BHP (tert‐butyl hydroperoxide) for 3 h. After discarding the culture medium, 100 μL of DMEM containing 10 μL of CCK‐8 solution was added to each well and incubated for an additional 2 h. Finally, the absorbance was measured at 450 nm using a Multiskan GO Microplate Spectrophotometer (Thermo Fisher Scientific, USA). Fifty micrograms per milliliter of Vitamin C (VC) was used as a positive control.

### 2.7 Measurement of Intracellular ROS Levels

HepG2 cells were cultured and treated with the same concentration of Y‐1 as in the cell viability assay. The key difference was that the cells were exposed to 0.4 mM *t*‐BHP for 30 min to induce ROS generation, followed by staining with 1 μM DCFH‐DA for 40 min. Fluorescence was measured using a fluorescence microplate reader with excitation/emission wavelengths of 485/525 nm (Zhang et al. 2020).

### 2.8 Cellular Antioxidant Activity of Y‐1

HepG2 cells were seeded at a density of 1.05 × 10^5^ cells/well in a 6‐well cell for 24 h. Subsequently, the cells were treated with Y‐1 (0–50 μg/mL) for 6 h, followed by exposure to 0.4 mM *t*‐BHP for 3 h (Hu et al. 2020; Wang et al. 2020). Fifty micrograms per milliliter of VC was used as a positive control.

Next, the cells were collected and lysed using an ultrasonic cell disruptor (200 W, ultrasound 3 s, interval 10 s, repeated 30 times), then centrifuged at 8000× *g* at 4°C for 10 min. The supernatants were immediately collected and stored on ice for subsequent analysis. The activities of antioxidant enzymes, including SOD, CAT, and GSH‐Px, as well as MDA levels, were measured using commercially available kits according to the manufacturer's instructions.

### 2.9 Analysis of Monosaccharide Composition

The monosaccharide composition of Y‐1 was determined using a Thermo ICS 5000+ ion chromatography system equipped with a Dionex CarboPac PA20 column (150 × 3.0 mm, 10 μm). Y‐1 was hydrolyzed with 2 M trifluoroacetic acid (TFA) at 121°C for 2 h and then dried under a stream of nitrogen. The residue was dissolved in methanol and repeatedly dried to remove excess TFA. Finally, the sample was dissolved in sterile water for high‐performance anion‐exchange chromatography (HPAEC) analysis. The monosaccharide standards were processed and analyzed in parallel with the samples.

### 2.10 Methylation Analysis

The polysaccharide Y‐1 sample was dissolved in DMSO and methylated using a DMSO/NaOH solution with CH3I (Yang et al. 2021). After complete methylation, the products were hydrolyzed with 2 M TFA at 121°C for 1.5 h, reduced with NaBD4, and acetylated with acetic anhydride at 100°C for 2.5 h. The acetylated derivatives were then dissolved in chloroform and analyzed by GC–MS using an Agilent 6890A‐5977B system equipped with an Agilent BPX70 chromatographic column (30 m × 0.25 mm × 0.25 μm, SGE, Australia).

### 2.11 NMR Analysis

The sample was dissolved in 0.5 mL of D_2_O to achieve a final concentration of 40 mg/mL. One‐dimensional NMR spectra (^1^H NMR, ^13^C‐NMR, DEPT‐135) and two‐dimensional NMR spectra (COSY, HSQC, TOCSY, NOESY) were recorded at 25°C using a Bruker AVANCE NEO 500 MHz spectrometer (Bruker, Rheinstetten, Germany).

### 2.12 Data Analysis

The data were analyzed using *t*‐tests conducted with GraphPad Prism 6.0 software, and statistical significance was set at *p* < 0.05. All cell‐based experiments were replicated a minimum of six times, and the results are expressed as mean ± standard deviation (SD).

## 3 Results and Discussion

### 3.1 Single‐Factor Experimental Analysis and BBD‐RSM Methodology to Extract SubPs

**FIGURE 1:**
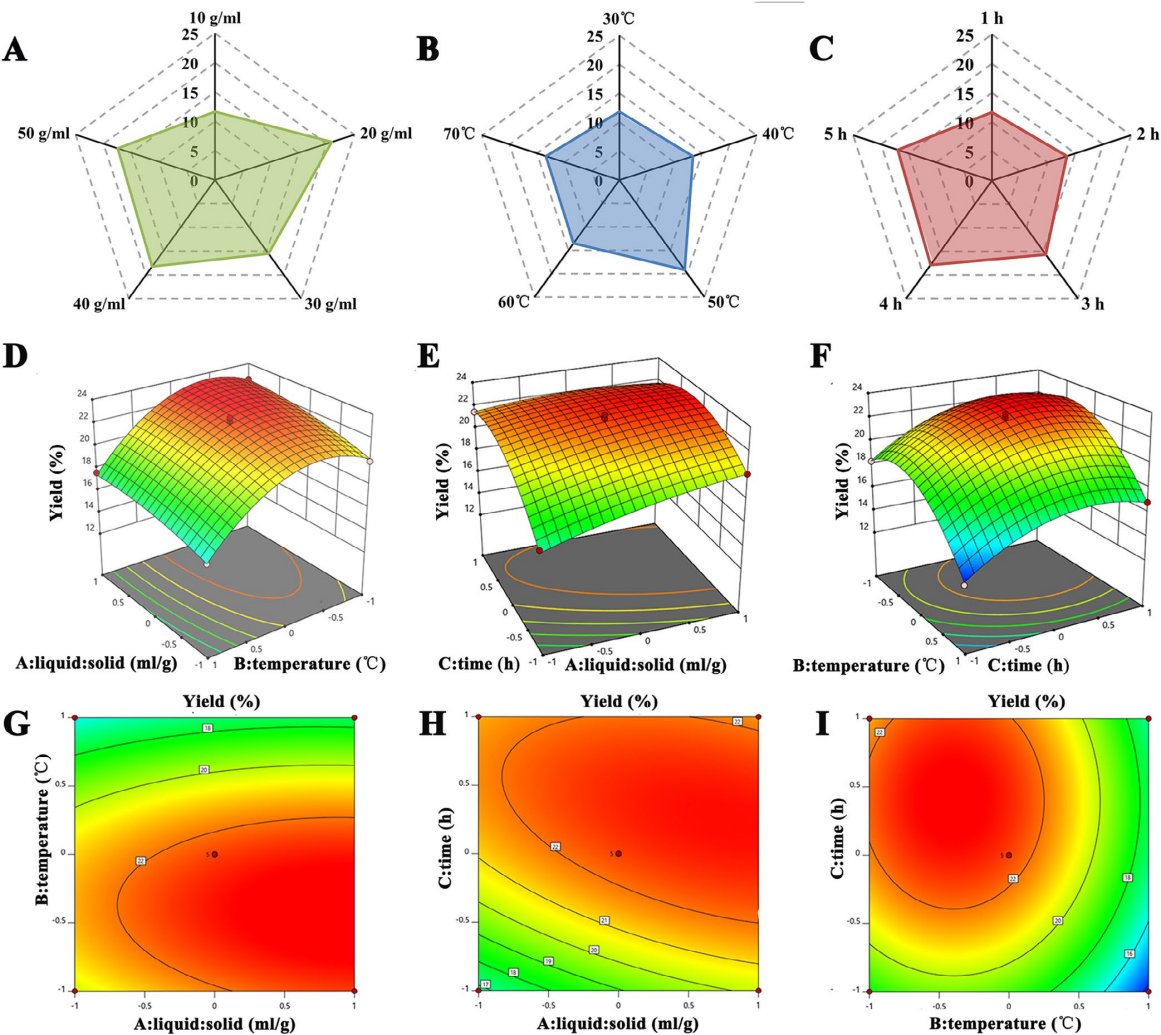
Single‐factor and response surface analysis of SubPs extraction yield. (A–C) Single‐factor effects of (A) liquid–solid ratio, (B) temperature, and (C) time. (D–I) Interaction effects: (D, G) ratio versus temperature; (E, H) ratio versus time; (F, I) time versus temperature.

The effects of liquid–solid ratio, extraction temperature, and extraction time on the yield of SubPs were investigated. As shown in Figure 1A, the polysaccharide extraction rate initially increased and subsequently decreased gradually with varying liquid–solid ratios. The extraction rate peaked at 20.93% ± 0.11% when the liquid–solid ratio was 20 mL/g.

The extraction yield increased as the temperature (Figure 1B) gradually rose from 30°C to 50°C, peaking at 19.18% ± 0.03% at 50°C. Subsequently, from 50°C to 70°C, the polysaccharide extraction rate decreased gradually.

As the extraction time increased (Figure 1C), the extraction yield of SubPs exhibited a linear upward trend during the first 1–4 h, reaching its maximum value of 17.86% ± 0.09% at 4 h. Prolonged extraction time and high temperatures may have damaged the structure of the polysaccharides (Liu et al. 2018), which could explain the observed decrease in yield.

Based on the results of single‐factor experiments, the central extraction conditions were determined to be a solid‐to‐liquid ratio of 1:20 g/mL, an extraction temperature of 50°C, and an extraction time of 4 h. The response surface analysis conducted around this central point (Table S1), with a 3‐factor 3‐level design, is summarized in Table S2. Through regression fitting analysis, the following multiple quadratic regression equation was derived:

$$\begin{array}{cc} Y=\; & 22.46+0.9106A-2.33B+1.56C-0.2223\mathrm{AB}-0.7895\mathrm{AC}\\ & -0.0265\mathrm{BC}-0.4804A^{2}-2.93B^{2}-1.97C^{2} \end{array}$$

The analysis of variance (ANOVA) for the regression model is presented in Table S3. The regression relationship of this model is highly significant (*p* < 0.01), while the lack‐of‐fit term (*p* = 0.7129 > 0.05) is not significant, indicating that the relationship between independent variables and response values is robust. The three‐dimensional response surfaces and contour plots are illustrated in Figure 1D–F. Analysis of these plots revealed that the optimal conditions for polysaccharide extraction were a liquid‐to‐solid ratio of 28 mL/g, an extraction temperature of 46°C, and an extraction time of 4 h, resulting in a maximum predicted yield of crude polysaccharide of 23.53%. The extraction process was validated under optimal conditions, yielding a polysaccharide extraction rate of 22.29% ± 0.44%, showing good agreement with the theoretical prediction.

### 3.2 Purification of SubPs

**FIGURE 2:**
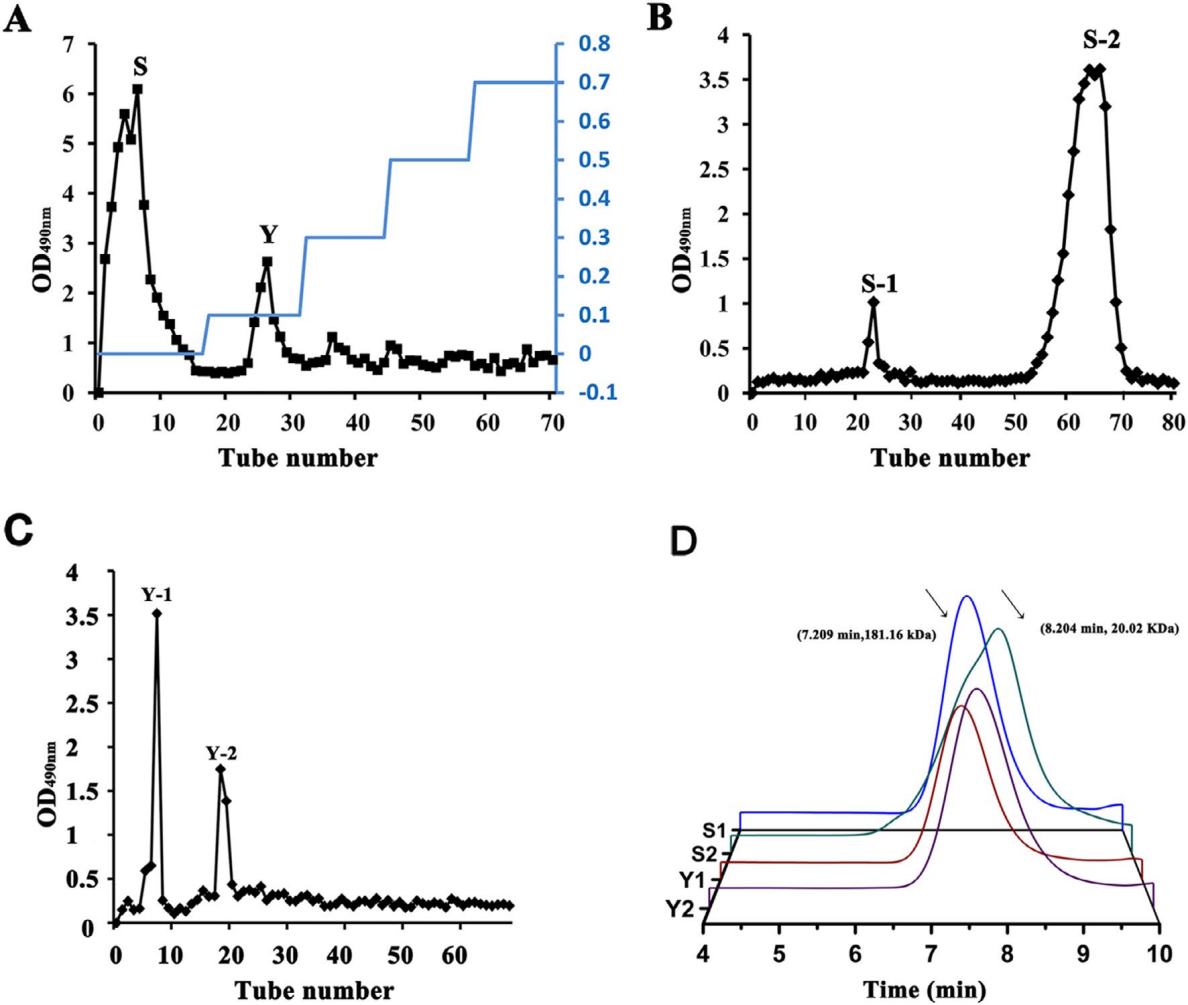
DEAE cellulose‐52 anion‐exchange (A), Sephadex G‐100 size exclusion column chromatography (B, C) curve of crude polysaccharide from *S. bovines*, and gel permeation chromatogram (D) of S‐1, S‐2, Y‐1, and Y‐2. The arrows point to the standard elution time and molecular weight.

SubPs was separated using a DEAE cellulose‐52 column (Figure 2A), resulting in the collection of two main elution peaks. The first peak, designated as S, was eluted with distilled water, while the second peak, named as Y, was eluted with 0.1 M NaCl. Subsequently, the S fraction was further purified using Sephadex G‐100 size exclusion column chromatography, yielding two main elution peaks, named S‐1 and S‐2 (Figure 2B). Similarly, another fraction Y also produced two main elution peaks, designated as Y‐1 and Y‐2 (Figure 2C). All four components (S‐1, S‐2, Y‐1, and Y‐2) were separately collected, concentrated, and freeze‐dried for further analysis.

The molecular weights of natural polysaccharides are correlated with their bioactivities (Li et al. 2016; Lin et al. 2020). Therefore, the molecular weights of S‐1, S‐2, Y‐1, and Y‐2 were investigated and compared. As shown in Figure 2D, Y‐1 exhibited the highest molecular weight (97.07 kDa), followed by S‐1 (61.46 kDa), Y‐2 (54.13 kDa), and S‐2 (47.34 kDa).

### 3.3 Antioxidant Effects of Y‐1 in *t*‐BHP‐Treated HepG2 Cells In Vitro

#### 3.3.1 Y‐1 Protects HepG2 Cells From *t*‐BHP‐Induced Cytotoxicity

**FIGURE 3:**
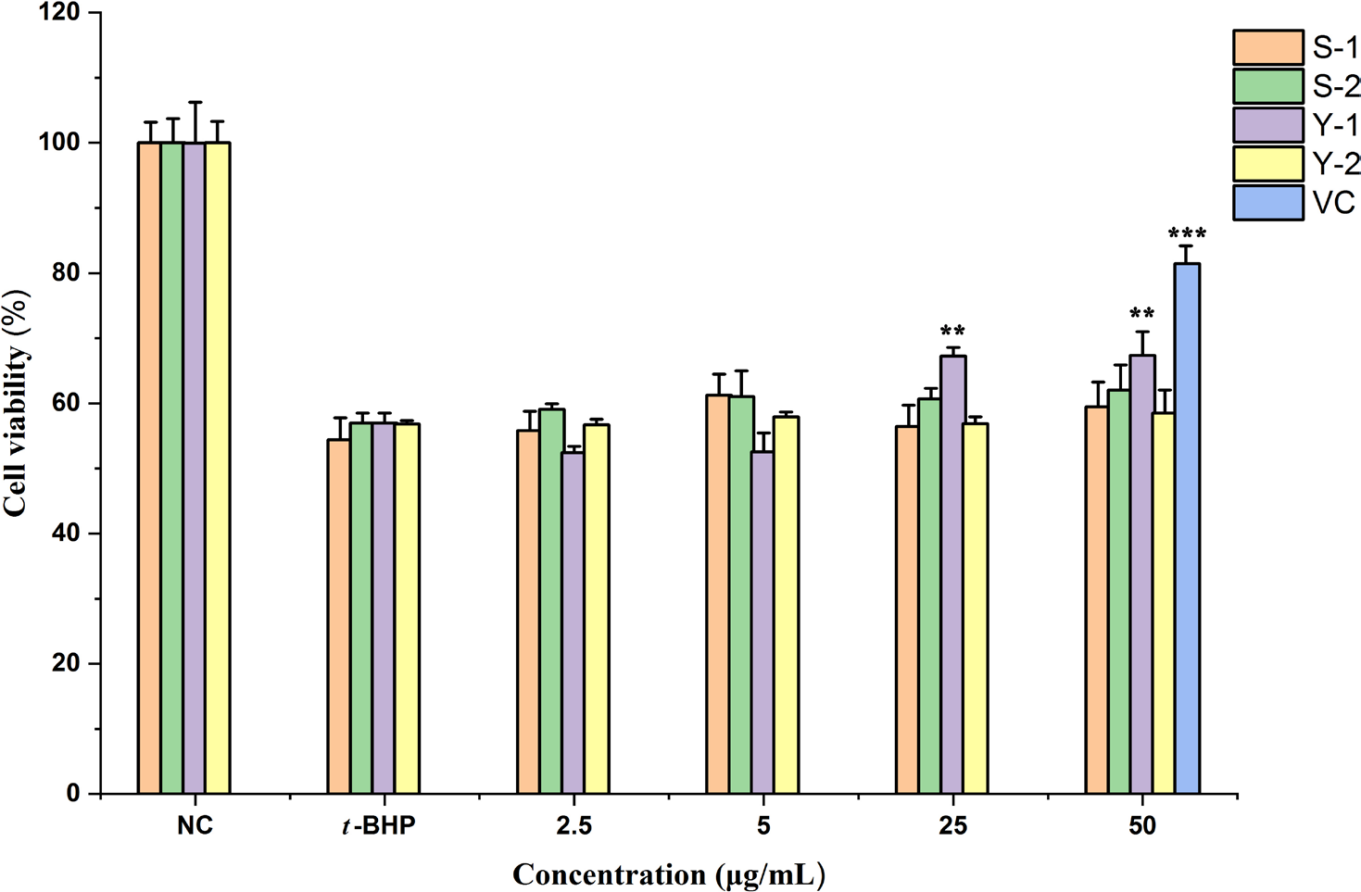
Effects of S‐1, S‐2, Y‐1, Y‐2, and VC on oxidative stress cells. **p < 0.01, ***p < 0.001 versus *t*‐BHP treated group; NC, none treated control group.

As shown in Figure 3, among the four polysaccharide components of SubPs, only Y‐1 demonstrated significant antioxidant activity, although its activity at 50 μg/mL remained lower than that of VC. Treatment with *t*‐BHP (400 μM) significantly inhibited the survival of HepG2 cells, reducing the cell survival rate to 56.98% ± 1.27%. With increasing concentrations of Y‐1 pretreatment, the survival rate of HepG2 cells gradually recovered. At concentrations of 25 and 50 μg/mL, the survival rate increased to over 65%, which was significantly different from the *t*‐BHP treatment group (*p* < 0.01).

The molecular weight of polysaccharides critically influences their bioactivity, with distinct weight ranges exhibiting varying biological effects (Xia et al. 2025). It modulates both physical properties and biological functions, where optimal distributions enhance solubility, stability, and bioavailability. Higher molecular weight polysaccharides typically demonstrate stronger activity due to increased active sites (Guo et al. 2025). The superior antioxidant activity of Y‐1 may be attributed to its specific structural characteristics, particularly its unique molecular weight distribution and monosaccharide composition.

#### 3.3.2 Effects on *t*‐BHP‐Induced Oxidative Stress Markers

Accumulation of ROS causes oxidative stress on cellular metabolism and can lead to the development of many diseases, such as heart disease, gastrointestinal inflammation, and cancer (Zhou et al. 2023). Therefore, we investigated the ROS scavenging ability of Y‐1.

**FIGURE 4:**
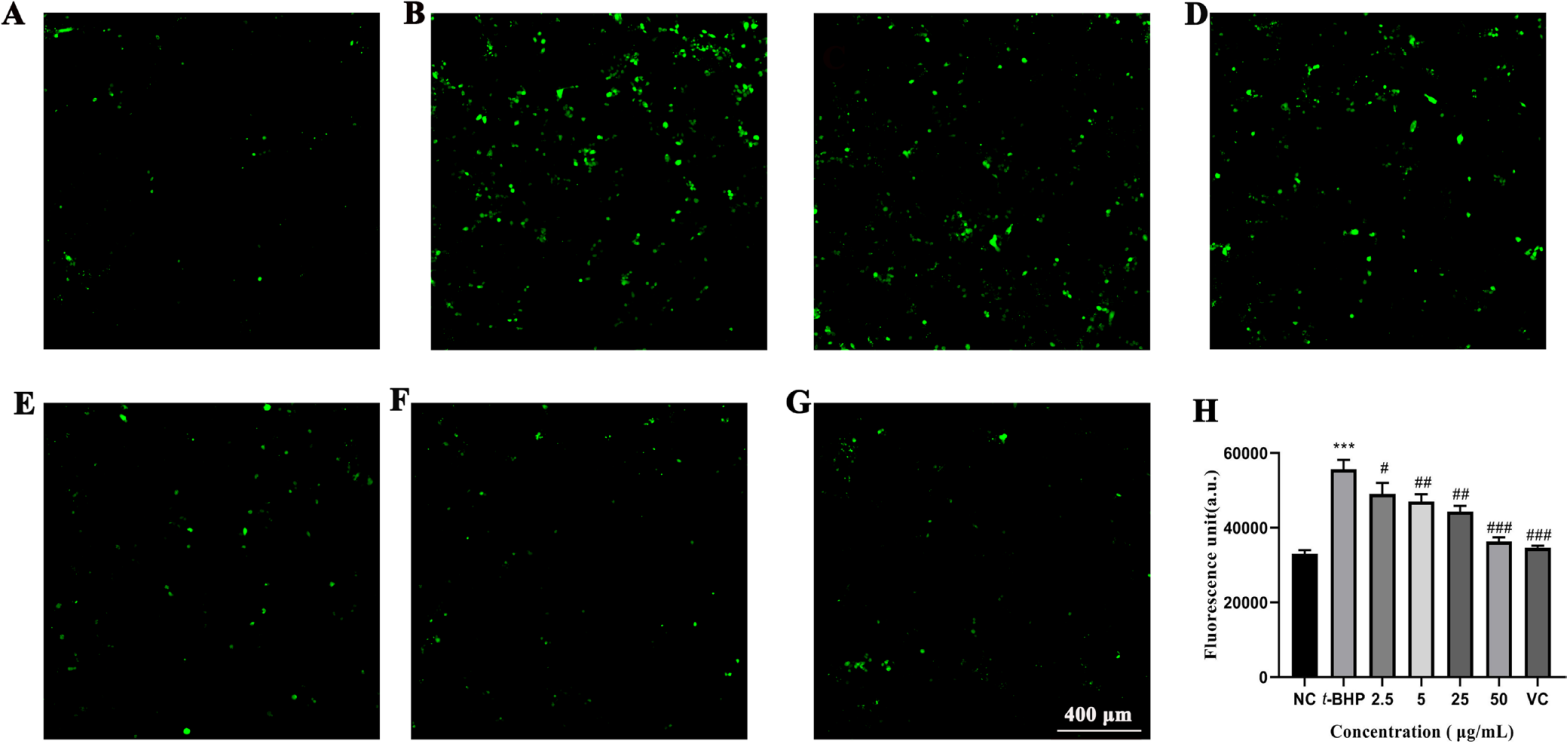
Scavenging ability of Y‐1 on ROS. (A) NC group. (B) *t*‐BHP‐treated group. (C–F) The group of 2.5, 5, 25, and 50 μg/mL Y‐1 pretreatment. (G) The positive control group of 50 μg/mL VC pretreatment. (H) The relative intensity of fluorescence in different groups. ***p < 0.001 versus the NC group; ^#^ *p* < 0.05, ^##^ *p* < 0.01, ^###^ *p* < 0.001 versus the *T*‐*BHP* group; NC, none treated control group.

As shown in Figure 4A–G, while the untreated control group displayed minimal fluorescence, the *t*‐BHP‐treated group exhibited intense fluorescence signals. Notably, Y‐1 treatment attenuated fluorescence intensity in a concentration‐dependent manner compared to the *t*‐BHP‐treated group, demonstrating its oxidative stress‐modulating capacity. Notably, at a concentration of 50 μg/mL, the protective efficacy of Y‐1 pretreatment was comparable to that of the positive control VC. The ROS levels in HepG2 cells were 1.58‐fold higher than those in untreated control cells (Figure 4H). At 50 μg/mL, Y‐1 reduced ROS levels by 34.73% ± 1.31% compared to the *t*‐BHP‐treated model group (*p* < 0.01). These results indicate that Y‐1 pretreatment significantly reduces ROS production in HepG2 cells under oxidative stress conditions.

In the antioxidant enzyme protection system, SOD, GSH‐Px, and CAT play crucial roles in scavenging free radicals and reducing or eliminating oxidative damage (Wang et al. 2023), while MDA is a product of lipid peroxidation and serves as a biomarker for oxidative stress (Liu et al. 2012). We investigated whether Y‐1 can enhance the activity of antioxidant enzymes and reduce MDA content in cells.

**FIGURE 5:**
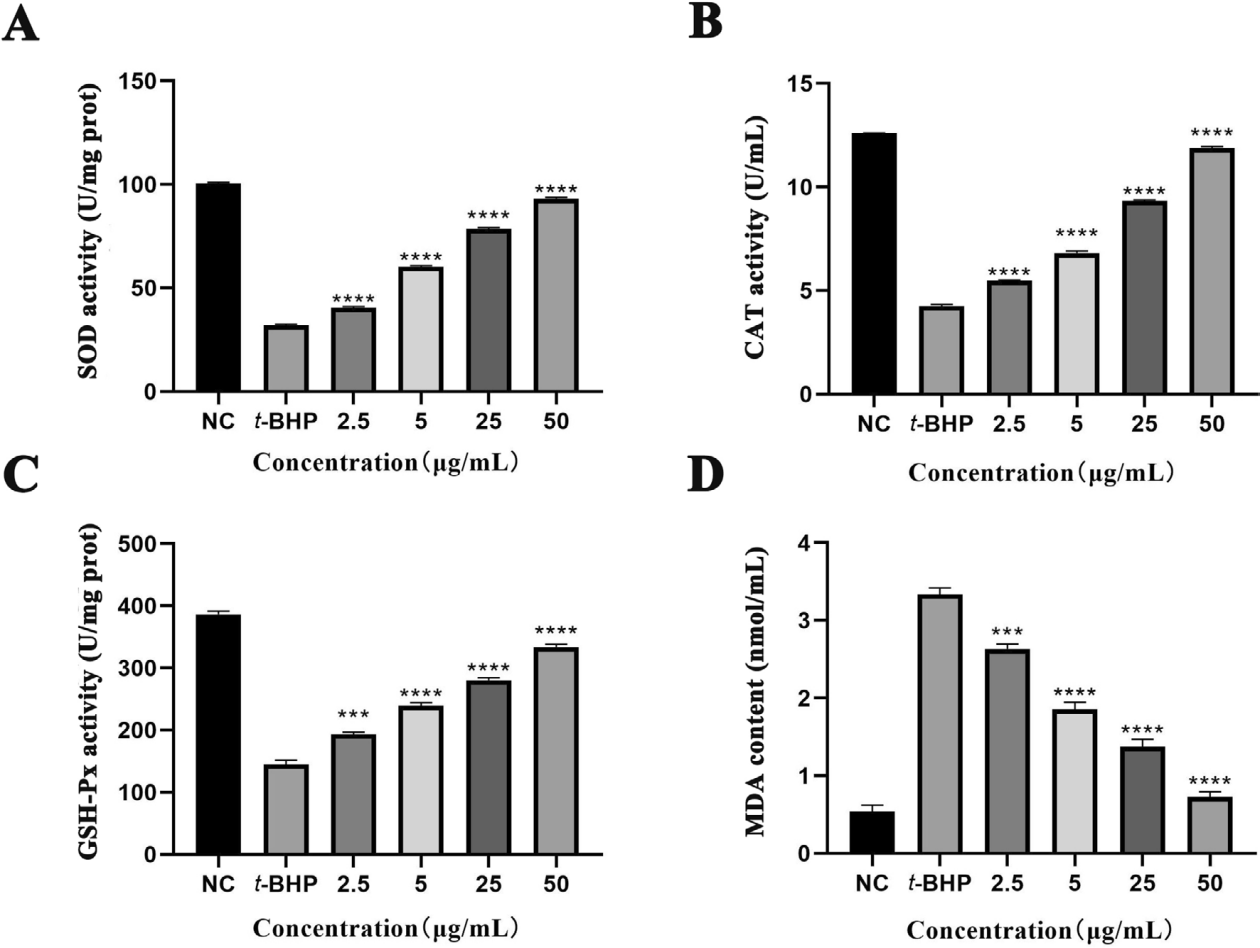
Effects of Y‐1 on the antioxidant system in *t‐*BHP‐treated HepG2 cells (*X* ± SD, *n* = 6): (A) SOD; (B) CAT; (C) GSH‐PX; and (D) MDA. ***p < 0.001, ****p < 0.0001 versus the *t*‐BHP group; NC, none treated control group.

As shown in Figure 5A–C, compared with the NC group, the activities of SOD, CAT, and GSH‐Px in the *t*‐BHP group were significantly decreased, to approximately 31.98 ± 0.56 U/mg prot, 4.26 ± 0.07 U/mL, and 144.75 ± 6.68 U/mg prot, respectively. However, with the gradual increase in Y‐1 treatment concentration, the activities of SOD, CAT, and GSH‐Px in cells significantly increased. When the concentration of Y‐1 reached 50 μg/mL, the activities of these three enzymes were restored to 93.22 ± 0.54 U/mg prot, 11.88 ± 0.08 U/mL, and 333.87 ± 4.31 U/mg prot, respectively, comparable to those of the NC group. These results indicate that Y‐1 exhibits strong antioxidant activity.

At the same time, the change in MDA content is also concentration‐dependent (Figure 5D). After treatment with *t*‐BHP, the MDA content significantly increased to 3.34 ± 0.09 nmol/mL. With the gradual increase in Y‐1 concentration, the MDA content gradually decreased. When the Y‐1 concentration reached 50 μg/mL, it was reduced to 0.73 ± 0.07 nmol/mL, which is approximately 1.34 times lower than that in the NC group.

Y‐1 exhibits potent antioxidant activity by significantly reducing ROS levels (34.73%) and restoring key antioxidant enzymes (SOD, CAT, GSH‐Px) in t‐BHP‐treated HepG2 cells, while markedly decreasing MDA content. These findings suggest Y‐1 could effectively counteract oxidative stress, making it a promising candidate for preventing oxidative damage‐related diseases. The heteropolysaccharide SGP2‐1 from *S. granulatus* enhanced macrophage functions (pinocytosis, ROS generation, cytokine release) through TLR2‐mediated MAPK/PI3K/Akt/NF‐κB activation (Gao et al. 2022), while *S. luteus* polysaccharide SLAP promoted CD4^+^/CD8^+^ T cell responses via gut microbiota‐bile acid modulation, ultimately suppressing tumor growth (Gao et al. 2024). Whether polysaccharides from *S. bovinus* exhibit similar bioactivities warrants further investigation.

### 3.4 Analysis of Monosaccharide Composition

**FIGURE 6:**
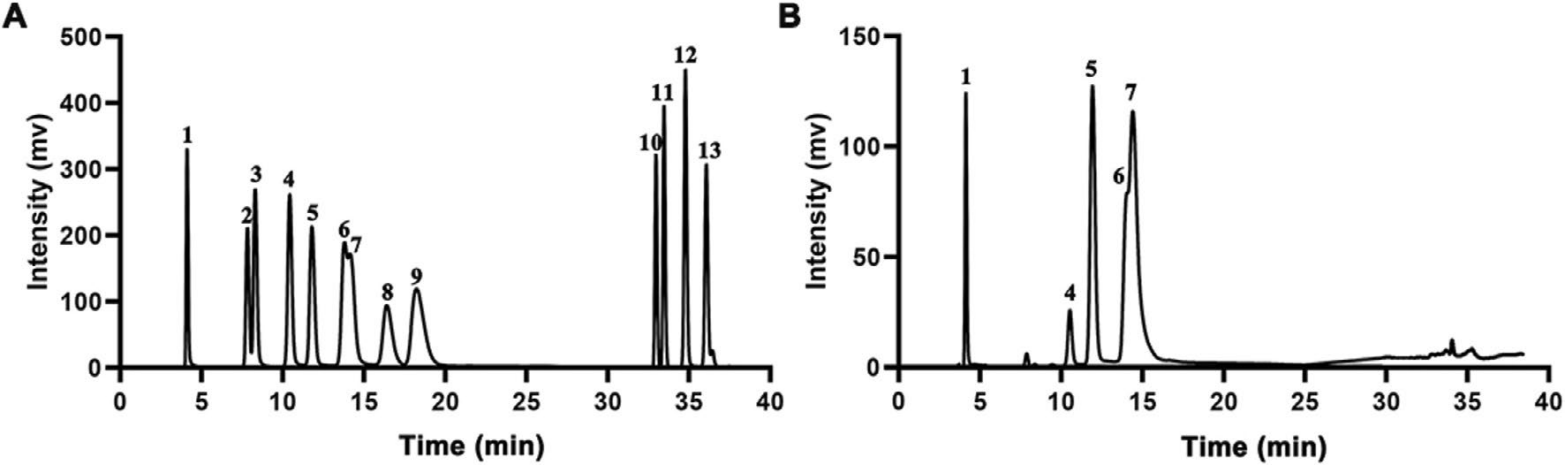
HPAEC chromatogram of monosaccharide composition: (A) mixed monosaccharide standard. Peaks 1–13 represent fucose, rhamnose, arabinose, galactose, glucose, xylose, mannose, fructose, ribose, galacturonic acid, glucuronic acid, mannuronic acid, and guluronic acid. (B) Monosaccharide composition of Y‐1.

Given the strong antioxidant properties of Y‐1, we conducted a more comprehensive analysis of its monosaccharide composition. The monosaccharide composition analysis serves as the foundation for examining the primary structure of polysaccharides and is presented in Figure 6A,B. The main monosaccharide components of Y‐1 include fucose (13.21%), galactose (3.54%), glucose (24.17%), xylose (15.37%), and mannose (40.78%). Mannose accounts for the highest proportion, followed by glucose. Previous research has demonstrated that mannose in polysaccharides contributes to antioxidant activity and is positively correlated with its content (Feng et al. 2021). The mannose content confers robust radical scavenging and reducing power abilities (Lo et al. 2011) and may enhance the activity of antioxidant enzymes (Shen et al. 2025). This may be due to the presence of functional hydroxyl groups in the mannose and molecules, which can donate electrons to neutralize free radicals (Trabelsi et al. 2021).

### 3.5 Methylation Analysis

To obtain more information about the linkage structure, GC**–**MS was employed for the methylation analysis of Y‐1. As shown in Table 1, eight main glycosidic linkage components were identified.

**TABLE 1:** Linkage analysis of Y‐1.

Peak	RT (min)	Partially methylated alditol acetate (PMAA)	Mass fragments (*m*/*z*)	Linkage types	Molar ratio (%)
1	5.606	1,5‐di‐O‐acetyl‐6‐deoxy‐2,3,4‐tri‐O‐methyl fucitol	72, 89, 102, 115, 118, 131, 162, 175	t‐Fuc (p)	7.11
2	5.951	1,5‐di‐O‐acetyl‐2,3,4‐tri‐O‐methyl xylitol	88, 101, 102, 118, 119, 161, 162	t‐Xyl (p)	6.57
3	7.124	1,5‐di‐O‐acetyl‐2,3,4,6‐tetra‐O‐methyl mannitol	87, 102, 118, 129, 145, 161, 162, 205	t‐Man (p)	9.73
4	7.200	1,5‐di‐O‐acetyl‐2,3,4,6‐tetra‐O‐methyl galactitol	87, 102, 118, 129, 145, 161, 162, 205	t‐Gal (p)	9.78
5	10.160	1,3,5‐tri‐O‐acetyl‐2,4,6‐tri‐O‐methyl mannitol	87, 101, 118, 129, 161, 202, 234	3‐Man (p)	27.36
6	11.940	1,4,5‐tri‐O‐acetyl‐2,3,6‐tri‐O‐methyl glucitol	87, 102, 113, 118, 129, 162, 233	4‐Glc (p)	11.14
7	12.708	1,2,3,5‐tetra‐O‐acetyl‐4,6‐di‐O‐methyl glucitol	87, 88, 101, 129, 161, 202, 262	2, 3‐Glc(p)	19.12
8	12.902	1,3,4,5‐tetra‐O‐acetyl‐2,6‐di‐O‐methyl mannitol	87, 118, 129, 143, 185, 203, 305	3, 4‐Man (p)	9.19

The results indicated that Y‐1 primarily contains 1,3‐linked mannose (27.36%), 1,4‐linked glucose (11.14%), 1,2,3‐linked glucose (19.12%), and 1,3,4‐linked mannose (9.19%). The terminal residues mainly consist of 1‐linked fucose (7.11%), 1‐linked xylose (6.57%), 1‐linked mannose (9.73%), and 1‐linked galactose (9.78%). While no degradation was detected under these conditions for our samples, temperature‐sensitive polysaccharides may benefit from milder protocols (Ciucanu and Kerek 1984).

### 3.6 NMR Analysis

**FIGURE 7:**
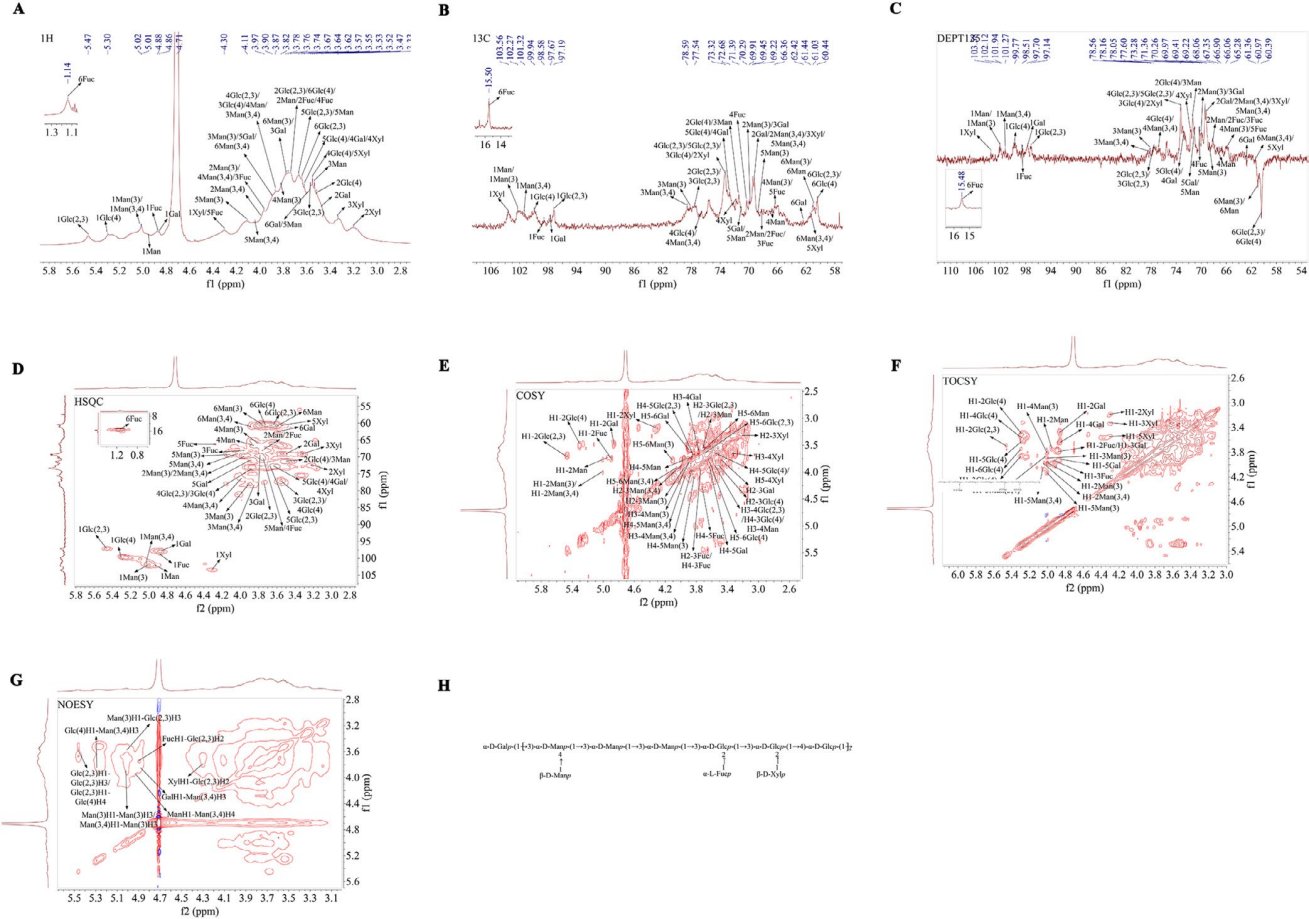
NMR spectra of Y‐1. (A) ^1^H NMR. (B) ^13^C NMR. (C) DEPT 135 NMR. (D) HSQC. (E) COSY. (F) TOCSY. (G) NOESY. (H) The partial primary structure of Y‐1.

The ^1^H NMR spectrum of Y‐1 (Figure 7A) shows proton signals distributed in the region of δ 3.0 to 5.5 ppm, with multiple signals identified in the anomeric signal region (4.3–5.4 ppm). The coupling signal peaks indicate the presence of various sugar residues. The chemical shifts corresponding to the anomeric proton signals are at 4.3, 4.86, 4.89, 4.94, 5.01, 5.02, 5.3, and 5.47 ppm. Non‐anomeric proton signals are primarily observed in the 3.1 to 4.2 ppm region. Notably, a strong signal peak at ~4.71 ppm corresponds to the solvent peak (Li et al. 2023).

In conjunction with ^13^C NMR (Figure 7B) and DEPT‐135 (Figure 7C) spectra, methylene signals at 60.74, 60.57, 60.37, 62.42, 61.03, 61.44, and 60.69 ppm were identified. Multiple signal peaks were observed in the heterogeneous carbon region of the sample. By correlating these with the cross‐peaks in the heteronuclear regions of the ^13^C NMR and HSQC spectra (Figure 7B,D), the anomeric signals of α or β‐glycosidic bond C‐1 were determined as follows: 5.01/102.04, 5.47/97.19, 5.3/99.94, 4.86/97.67, 4.94/102.27, 5.02/101.32, 4.89/98.58, and 4.3/103.56 ppm. These signals are designated as sugar residues A, B, C, D, E, F, G, and H, respectively.

In the COSY and TOCSY spectrum (Figure 7E,F), the residual proton information for sugar residues A to H was determined based on the overlapping peaks (Table 2). The chemical shifts of the carbons in these sugar rings were assigned using HSQC signals. Notably, the chemical shifts of C1 and C3 in sugar residue A shifted to lower fields, indicating substitution at the O‐1 and O‐3 positions of the sugar ring. This suggests that sugar residue A is likely →3)‐α‐D‐Manp‐(1→ (Li et al. 2023). The chemical shifts of C1, C2, and C3 in sugar residue B shifted to lower fields, indicating substitution at the O‐1, O‐2, and O‐3 positions of the sugar ring. This suggests that sugar residue B is likely →2, 3)‐α‐D‐Glcp‐(1→ (Zhu et al. 2021). The chemical shifts of C1 and C4 in the sugar residue C shifted towards lower fields, indicating that substitution has occurred at the O‐1 and O‐4 positions of the sugar ring, so the sugar C was →4)‐α‐D‐Glcp‐(1→ (Liu et al. 2022). Sugar residue D is identified as α‐D‐Galp‐(1→ (Kawahara et al. 2000). Sugar residue E was identified as β‐D‐Manp‐(1→ (Chen et al. 2020), sugar residue F as →3,4)‐α‐D‐Manp‐(1→ (Yang et al. 2022), sugar residue G as α‐L‐Fucp‐(1→ (Zhou et al. 2024), and sugar residue H as β‐D‐Xylp‐(1→ (Qian et al. 2012).

**TABLE 2:** The chemical shifts of ^1^H and ^13^C of each sugar residue.

Code	Glycosyl residues	Chemical shifts (ppm)					
		H1/C1	H2/C2	H3/C3	H4/C4	H5/C5	H6/C6
A	→3)‐α‐D‐Man*p*‐(1→	5.01	3.97	3.9	3.82	4.11	3.78
		102.04	69.71	78.03	66.39	68.85	60.74
B	→2,3)‐α‐D‐Glc*p*‐(1→	5.47	3.73	3.57	3.85	3.68	3.64
		97.19	77.5	77.32	73.08	73.16	60.57
C	→4)‐α‐D‐Glc*p*‐(1→	5.30	3.51	3.87	3.55	3.63	3.74
		99.94	71.26	73	76.94	72.84	60.37
D	α‐D‐Gal*p*‐(1→	4.86	3.48	3.77	3.63	3.89	3.59
		97.67	69.24	69.7	72.84	71.03	62.42
E	β‐D‐Man*p*‐(1→	4.94	3.73	3.53	3.86	3.71	3.59
		102.27	68.01	71.24	66.95	71.05	61.03
F	→3,4)‐α‐D‐Man*p*‐(1→	5.02	4.00	3.86	3.97	4.06	3.9
		101.32	69.53	78.59	76.62	69.05	61.44
G	α‐L‐Fuc*p*‐(1→	4.89	3.75	3.95	3.73	4.29	1.14
		98.58	68.1	68.22	70.7	66.32	15.5
H	β‐D‐Xyl*p*‐(1→	4.30	3.20	3.33	3.63	3.55	nd
		103.56	73.94	69.24	71.8	60.69	nd

Abbreviation: nd: not detected.

The structural and linkage characteristics of the polysaccharide were elucidated based on the chemical shifts of ^13^C and ^1^H for each sugar residue in the sample, as well as the analysis of NOESY (Figure 7G) spectra to infer the connectivity of the sugar residues. According to the NOESY data, cross‐peaks were observed between H1 and H3 of sugar residue A (δ 5.01/3.9 ppm), H1 of sugar residue A and H3 of sugar residue B (δ 5.01/3.57 ppm), H1 and H3 of sugar residue B (δ 5.47/3.57 ppm), H1 of sugar residue B and H4 of sugar residue C (δ 5.47/3.55 ppm), H1 of sugar residue C and H3 of sugar residue F (δ 5.3/3.86 ppm), H1 of sugar residue D and H3 of sugar residue F (δ 4.86/3.86 ppm), H1 of sugar residue E and H4 of sugar residue F (δ 4.94/3.97 ppm), H1 of sugar residue F and H3 of sugar residue A (δ 5.02/3.9 ppm), H1 of sugar residue G and H2 of sugar residue B (δ 4.89/3.73 ppm), and H1 of sugar residue H and H2 of sugar residue B (δ 4.3/3.73 ppm).

Consequently, integrating one‐dimensional and two‐dimensional nuclear magnetic resonance (NMR) data with methylation analysis, it was inferred that the polysaccharide backbone (Figure 7H) is primarily composed of →3)‐α‐D‐Manp‐(1→, →2,3)‐α‐D‐Glcp‐(1→, →4)‐α‐D‐Glcp‐(1→, and →3,4)‐α‐D‐Manp‐(1→ units. The side chains are mainly constituted by β‐D‐Manp‐(1→ linked at the O‐4 position of →3,4)‐α‐D‐Manp‐(1→ and α‐L‐Fucp‐(1→ or β‐D‐Xylp‐(1→ attached to the O‐2 position of →2,3)‐α‐D‐Glcp‐(1→. Additionally, a small amount of α‐D‐Galp‐(1→ may be present at the non‐reducing end, linked at the O‐3 position of →3,4)‐α‐D‐Manp‐(1→. This heteropolysaccharide has a complex structure, and its unique glycosidic bonds—particularly the →2,3)‐α‐D‐Glc(p) linkage—warrant further investigation to elucidate their contribution to antioxidant activity.

## 4 Conclusion

This study optimized the extraction of *S. bovinus* polysaccharides, yielding four fractions (S‐1, S‐2, Y‐1, Y‐2). Among these, Y‐1 (97.07 kDa) demonstrated superior antioxidant activity, effectively protecting HepG2 cells against oxidative stress by reducing ROS levels, enhancing antioxidant enzyme activities (SOD/CAT/GSH‐Px), and decreasing MDA content. Structural analysis revealed Y‐1's unique composition, featuring a backbone mainly connected by →3)‐α‐D‐Manp‐(1→, →2,3)‐α‐D‐Glcp‐(1→, →4)‐α‐D‐Glcp‐(1→, and →3,4)‐α‐D‐Manp‐(1→. Branching occurs primarily via β‐D‐Manp‐(1→ at the O‐4 position and α‐L‐Fucp‐(1→ or β‐D‐Xylp‐(1→ attached to the O‐2 position of →2,3)‐α‐D‐Glcp‐(1→. Additionally, there may be a small amount of α‐D‐Galp‐(1→ attached to the O‐3 position of →3,4)‐α‐D‐Manp‐(1→ at the non‐reducing end. The remarkable bioactivity of Y‐1 is likely attributed to its high molecular weight and distinctive structure, positioning it as a promising natural antioxidant worthy of further development. In subsequent studies, we will investigate its structure–activity relationship to elucidate the antioxidant mechanism of Y‐1.

## Author Contributions

**Zhuang Li:** data curation (lead), investigation (equal), methodology (equal). **Shuting Fan:** data curation (equal), investigation (equal), methodology (equal). **Xueqi Wang:** data curation (equal), investigation (equal), methodology (equal). **Li‐an Wang:** funding acquisition (equal), supervision (equal). **Jianhua Lv:** methodology (equal), writing – original draft (equal), writing – review and editing (equal). **Jinxiu Zhang:** data curation (equal), investigation (lead), writing – review and editing (lead), funding acquisition (equal).

## Supporting information


**Table S1:** fsn370995‐sup‐0001‐Tables.docx.

## Data Availability

The data that support the findings of this study are available on request from the corresponding author.
