# Supplementary material for: Extraction Optimization and Characterization of Antioxidant Polysaccharides From Suillus bovinus
Source: Food Sci Nutr. 2025 Sep 19;13(9):e70995. doi: 10.1002/fsn3.70995 (PMC12447242; doi:10.1002/fsn3.70995)
Supplement: Supplementary file 1 — Table S1: fsn370995‐sup‐0001‐Tables.docx. [file FSN3-13-e70995-s001.docx]

**Extraction optimization and characterization of *Suillus bovinus* polysaccharide with and antioxidant activities**

Table S1 Factors and levels of BBD

| Level | Factor | | |
| --- | --- | --- | --- |
|  | A:liquid–solid（mL/g） | B:temperature（℃） | C:Time（h） |
| -1 | 10 | 40 | 3 |
| 0 | 20 | 50 | 4 |
| +1 | 30 | 60 | 5 |

Table S2 Results of Box-Behnken design

| Run numbers | A:liquid–solid（mL/g） | B:temperature（℃） | C:Time（h） | Extraction yield (%) | |
| --- | --- | --- | --- | --- | --- |
|  |  |  |  | Experimental values | Predicted  values |
| 1 | -1 | 0 | -1 | 17.00 | 16.75 |
| 2 | -1 | -1 | 0 | 20.05 | 20.25 |
| 3 | 0 | -1 | -1 | 18.27 | 18.31 |
| 4 | 0 | 0 | 0 | 22.75 | 22.46 |
| 5 | 0 | 0 | 0 | 22.09 | 22.46 |
| 6 | 1 | 0 | 1 | 21.46 | 21.70 |
| 7 | 1 | 1 | 0 | 17.62 | 17.42 |
| 8 | -1 | 0 | 1 | 21.44 | 21.46 |
| 9 | 0 | 1 | 1 | 16.82 | 16.78 |
| 10 | 0 | -1 | 1 | 21.70 | 21.48 |
| 11 | 1 | 0 | -1 | 20.17 | 20.16 |
| 12 | 0 | 1 | -1 | 13.49 | 13.70 |
| 13 | -1 | 1 | 0 | 16.01 | 16.04 |
| 14 | 1 | -1 | 0 | 22.54 | 22.51 |
| 15 | 0 | 0 | 0 | 23.08 | 22.46 |
| 16 | 0 | 0 | 0 | 22.40 | 22.46 |
| 17 | 0 | 0 | 0 | 22.01 | 22.46 |

Table S3. Analysis of variance (ANOVA) of the quadratic model and lack of fit.

| Source | Sum of Squares | Degree of freedom | Mean square | F-value | P-value |  |
| --- | --- | --- | --- | --- | --- | --- |
| Model | 129.72 | 9 | 14.41 | 91.07 | < 0.0001 | significant |
| A | 43.32 | 1 | 43.32 | 41.91 | 0.0003 |  |
| B | 6.63 | 1 | 6.63 | 273.73 | < 0.0001 |  |
| C | 19.53 | 1 | 19.53 | 123.40 | < 0.0001 |  |
| AB | 0.1976 | 1 | 0.1976 | 1.25 | 0.3007 |  |
| AC | 0.0028 | 1 | 0.0028 | 15.75 | 0.0054 |  |
| BC | 2.49 | 1 | 2.49 | 0.0177 | 0.8978 |  |
| A² | 36.14 | 1 | 36.14 | 6.14 | 0.0424 |  |
| B² | 0.9716 | 1 | 0.9716 | 228.36 | < 0.0001 |  |
| C² | 16.29 | 1 | 16.29 | 102.94 | < 0.0001 |  |
| Residual | 1.11 | 7 | 0.1583 |  |  |  |
| Lack of Fit | 0.2938 | 3 | 0.0979 | 0.4812 | 0.7129 | not significant |
| Pure Error | 0.8141 | 4 | 0.2035 |  |  |  |
| Cor Total | 130.83 | 16 |  |  |  |  |
